# Rare Recurrent EWSR1-PLAGL1 Rearranged Intracranial Tumor With Biphasic Epithelioid Differentiation: One Case Report With Literature Review

**DOI:** 10.3389/fonc.2022.938385

**Published:** 2022-07-14

**Authors:** Ai-yan Xing, Wen-wei Yang, Yu-lu Liu, Nan-nan Sun, Xiao-meng Hao, Su-xia Wang, Kun Mu

**Affiliations:** ^1^ Department of Pathology, Qilu Hospital of Shandong University, Jinan, China; ^2^ Department of Medical Oncology, National Cancer Center/National Clinical Research Center for Cancer/Cancer Hospital, Chinese Academy of Medical Sciences and Peking Union Medical College, Beijing, China; ^3^ Department of Pathology, The Affiliated Yantai Yuhuangding Hospital of Qingdao University, Yantai, China; ^4^ Department of Pathology, School of Basic Medical Sciences, Shandong University, Jinan, China

**Keywords:** EWSR1-PLAGL1 fusion, intracranial tumors, recurrence, biphasic differentiation, TERT

## Abstract

EWSR1-rearranged tumors encompass a rare and heterogeneous group of entities with features of the central nervous system (CNS) mesenchymal and primary glial/neuronal tumors. EWSR1-PLAGL1 gene fusion is a particularly rare form of rearrangement. We presented a recurrent intracranial EWSR1-PLAGL1 rearranged tumor and reviewed the relevant literature. In this case, histopathology and immunohistochemistry (IHC) were evaluated for both the primary and relapsed tumors. Fluorescence *in situ* hybridization (FISH) and next-generation sequencing (NGS) were performed for the relapsed tumor. We compared the morphology, IHC results and molecular features with the previously reported EWSR1-PLAGL1 rearranged CNS tumors. Our case exhibited a unique feature with a variable biphasic pattern of epithelioid differentiation, which differed from the two reported groups. The primary and relapsed tumors both expressed cytokeratin of the focal area with epithelioid differentiation. The recurrent tumor showed an increased proliferation index (average Ki-67 index of 15%) compared with the primary tumor (average Ki-67 index of 5%). NGS showed that TERT promoter mutation was the only molecular change besides EWSR1-PLAGL1 fusion. Our study provides further insight into intracranial tumors with EWSR1-PLAGL1 fusion, representing a distinct CNS tumor with no-reported histological and immunohistochemical features. Future studies, particularly for the biphasic differentiation and the role of TERT promoter mutation were needed to clarify this unusual chromosomal rearrangement in the CNS tumor.

## Introduction

In the past few decades, nucleotide-based methods, such as DNA and RNA sequencing and genome-wide analysis of DNA methylation, have been shown to assist in tumor diagnosis and classification. The fifth edition of the WHO Classification of Tumors of the Central Nervous System (WHO CNS 5th) has incorporated more molecular features into the classification of CNS tumors (1). Primary CNS EWSR1 gene fusion tumors are uncommon. The EWSR1-rearranged CNS tumors showed a broad spectrum of morphology and biologic behavior. Morphologically, those tumors can be categorized as mesenchymal tumors or glial/neuronal tumors. Specifically, EWSR1-PLAGL1 rearranged CNS tumors are extremely rare. Here, we report a recurrent intracranial EWSR1-PLAGL1 rearranged tumor differing from the previous reports with unique biphase epithelioid differentiation and immunohistochemistry features.

## Materials and methods

### Clinical History

**Figure 1:**
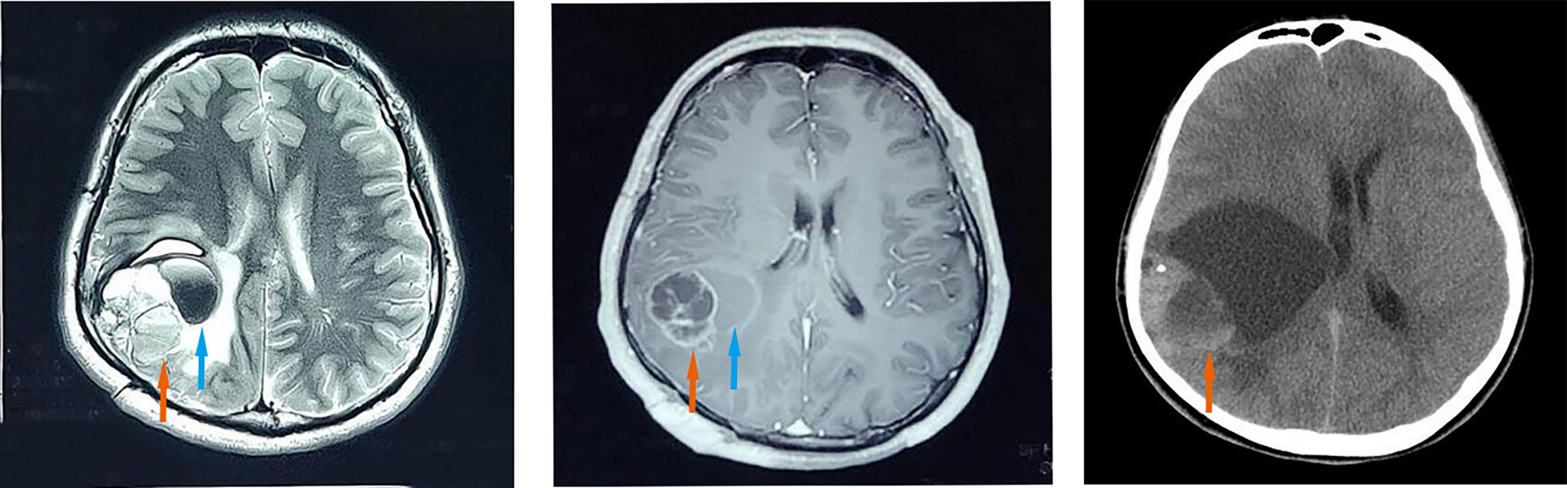
Imaging features. The recurrent lesion appeared as a mixed-signal intensity located in the right temporal-parietal lobe with a polycystic compartment, patchy hypointensity (orange arrow), liquid level inside (blue arrow), and line-like low signal areas at the periphery, surrounded by extensive oedema on axial T2-weighted images (left). Axial contrast-enhanced T1-weighted MR image (middle) demonstrated marked enhancement in the solid portions (orange arrow) of the recurrent mass and no enhancement of the cyst (blue arrow). Axial CT scan of the primary lesion showed a round-like area of mixed density (orange arrow), measuring about 6.3*6.3 cm, in the brain parenchyma of the right parietal lobe with compression and deformation of the right lateral ventricle (right).

A 26-year-old male presented with worsening headaches and vomited for two months. MRI of the brain revealed a mass at the right temporal-parietal lobe. T2WI showed a polycystic compartment-like change, and the area is approximately 5.4*4.3cm. A sheet-like low signal and liquid level were found on the inside. The surrounding brain is edema and compressed (**Figure 1**; left). Brain-enhanced CT (T1W1) showed a solid cystic enhancement of the mass (**Figure 1**; middle). The entire lesion was surgically removed. Ten years ago, the patient underwent right temporal-parietal tumor resection in a local hospital and was pathologically diagnosed with “atypical central neurocytoma”. CT of the primary lesion showed a round-like 6.3*6.3 cm mixed density shadow in the brain parenchyma of the right parietal lobe with a compressed and deformed ventricle (**Figure 1**; right). No radiotherapy or chemotherapy was received after the initial surgery. A routine brain scan was performed annually until three years before the recurrence. The patient received a radiotherapy (54Gy) after recurred tumor resection. The whole-body imaging scan found no other lesions, and the patient is in good condition currently.

### Histopathology and Immunohistochemistry (IHC)

5μm formalin-fixed paraffin-embedded sections were used for hematoxylin & eosin (H&E) and IHC stained. IHC was performed by an automatic immunohistochemical staining instrument (Ventana BenchMark ULTRA). The primary antibodies of GFAP (MX047), Vimentin (MX034), broad-spectrum Cytokeratin (AE1/AE3), Desmin (MAB-0766), and EMA (E29) were from MXB Biotechnologies. Commercially available prediluted antibodies of ZSGB-BIO, including Syn (EP158), S100 (ZA-0225), CgA (LK2H10), NSE (5E2), NeuN (A60), HMB45 (ZM-0187), Nestin (EP287), Oligo-2 (EP112), INI-1(ZA-0696), Neurofilament (ZM-0198) and Ki67 (UMAB107) were used. Photographs were captured using an Olympus BX51 microscope and an Olympus SC180 camera.

### Fluorescence *In Situ* Hybridization (FISH)

FISH for EWSR1 (22q12) rearrangement was performed on 4μm paraffin sections using the Vysis LSI EWSR1 dual-color break-apart probe (Abbott Molecular, Des Plaines, IL) according to standard protocols. The probe to the 5’ EWSR1 sequence is labeled in green and the probe to the 3’ EWSR1 sequence is labeled in red. A 4’,6-diamidino-2-phenylindole (DAPI) counterstain was used to identify the tumor cell nuclei. Analysis was performed on an epifluorescence microscope using single interference filter sets for red and green. For each interference filter, monochromatic images were acquired and merged using CytoVision (Leica Microsystems). A specimen was considered positive for EWSR1 rearrangement when a minimum of 15% of analyzed cells displayed split red and green signals or a single signal.

### Next-Generation Sequencing (NGS)

Tumor genomic DNA was extracted from FFPE samples using QIAamp DNA FFPE Tissue Kit. Libraries were constructed using the KAPA Hyper DNA Library Prep Kit (KAPA Biosystem, KK8504). The probes (Nanjing Geneseeq Biotechnology) for targeted sequencing cover exons, selected introns and alternatively spliced regions of 82 glioma-related genes (see **Supplementary Table**). After hybridization, capture and purification, the library was sequenced as paired 150-bp reads on Illumina HiSeq 4000 according to the manufacturer’s instrument. Base-calling was performed on bcl2fastq v2.16.0.10 (Illumina, Inc.) to generate sequence reads in FASTQ format (Illumina 1.8+ encoding). High-quality reads were mapped to the human genome (hg19, GRCh37 Genome Reference Consortium Human Reference 37) using the BWA aligner 0.7.12 with the BWA-MEM algorithm and default parameters to create SAM files. The Genome Analysis Toolkit (GATK, version 3.4-0) was used to locally realign the BAMs files at intervals with indel mismatches and recalibrate base quality scores of reads in BAM files. The combined fusion results from all tools were manually reviewed on IGV for confirmation.

## Results

### Microscopic Features

**Figure 2:**
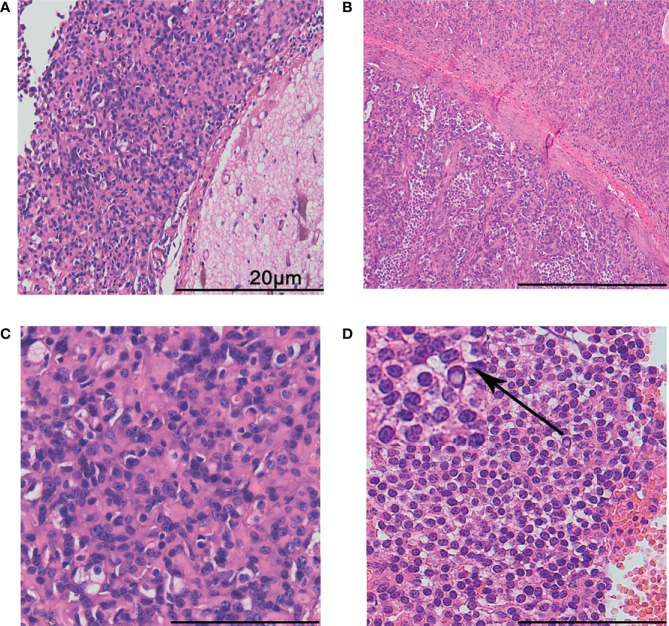
Histological features. The boundary between the tumor and the surrounding brain tissues was distinct **(A)**. Fibrous segmentations were seen inside the tumor. The lower left part presented with more epithelioid tumor cell islands **(B)**. Most tumor cells were ovally arranged in thick cords with unclear cell boundaries, scattered with few vacuolated cytoplasm cells **(C)**. Epithelioid cell islands were uniform, round with clear cell membrane **(D)**. The arrow shows a magnification of the cells in upper left corner. The scale bar represents 20 μm.

Both primary and recurrent tumors showed distinct boundaries between the tumor areas and the surrounding brain parenchyma (**Figure 2A**). Fibrous segmentations were seen inside the tumor (**Figure 2B**). There were two distinct tumor cell types: most were oval with unclear cell boundaries and scattered with few vacuolated cytoplasm cells (**Figure 2C**). In focal areas, enlarged epithelioid cell islands were seen with monomorphic, round nuclei, speckled nuclear chromatin, vacuolated cytoplasm, and distinct membrane (**Figure 2D**; upper left corner; arrow means magnification). No myxoid, vascular endothelial cell proliferation, calcifications, or necrosis was found in the primary and recurrent tumors. These tumors were well-circumscribed, and the tumor cells were mild and surrounded thin-walled blood vessels. However, increased and enlarged epithelioid tumor cell islands were found in the recurrent tumor samples.

### Immunohistochemical Findings

**Figure 3:**
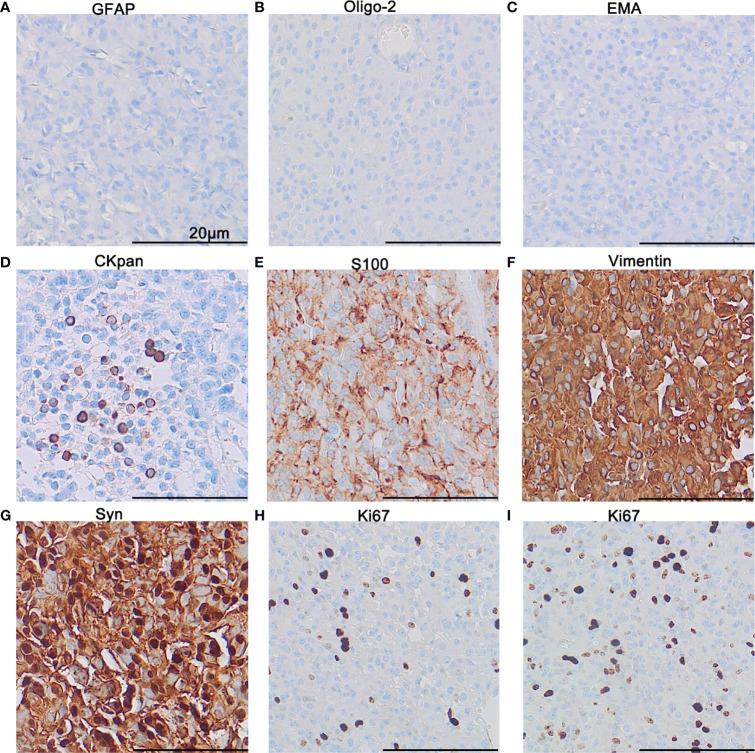
Immunohistochemistry. Immunohistochemical staining demonstrated negativity for GFAP **(A)**, Oligo-2 **(B)**, and EMA **(C)**, focal expression of CKpan **(D)** and patchy expression of S100 **(E)**, diffuse expression of Vimentin **(F)**, and Syn **(G)**. The proliferation index of Ki67 in the hot-spot area for the primary tumor **(H)** and the recurrent tumor **(I)**. The scale bar represents 20 μm.

All the tumor cells were negative for GFAP (**Figure 3A**), Oligo-2 (**Figure 3B**), and EMA (**Figure 3C**). Negative staining was also observed for NSE, NF, CgA, NeuN, Nestin, Desmin, HMB45, and INI-1(not shown). Epithelioid cell islands and scattered vacuolated cytoplasm cells showed positive expression for Cytokeratin (**Figure 3D**). S100 was positive in a patchy pattern (**Figure 3E**), and strong diffuse staining for Vimentin (**Figure 3F**) and Syn (**Figure 3G**) were found for all the tumor areas. Both the primary and the recurrent tumors showed the same immunophenotype including positive expression of Vimentin and Syn, negative for GFAP, Oligo-2, and EMA. However, more Cytokeratin positive epithelioid tumor cells and an increased Ki67 index were found in recurrent tumors. The average percentage of the Ki67 positive tumor cell is 5% for the primary tumor (**Figure 3H**), while 15% for the recurrent tumor (**Figure 3I**).

### Molecular Features

**Figure 4:**
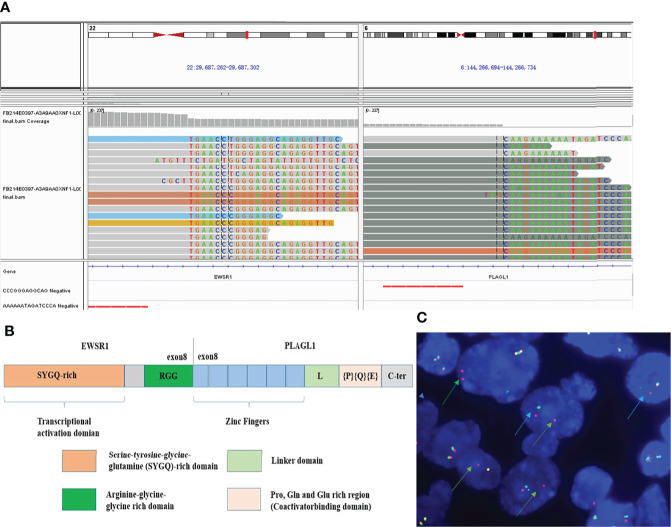
Molecular features. NGS analysis revealed EWSR1-PLAGL1 fusion **(A)**. The fusion site was at exon 8 of the EWSR1 and exon 8 of the PLAGL1 gene **(B)**. FISH showed positive EWSR1 gene rearrangement **(C)** using EWSR1 dual-color break-apart probes, with red-green separate (blue arrow) or individual red signal (green arrow).

NGS analysis revealed EWSR1-PLAGL1 fusion (**Figure 4A**) as well as TERT (c.-124C>T, see **Supplementary Figure**) promotor mutation. No other gene mutation (Indels or CNVs) was found. The fusion site is exon 8 of the EWSR1 gene and exon 8 of the PLAGL1 gene (**Figure 4B**). FISH with EWSR1 break-apart probe showed red-green separate (blue arrow) or individual red signal (green arrow) in more than 70% of tumor cells, which suggested positive EWSR1 gene rearrangements (**Figure 4C**).

## Discussion

EWSR1-rearranged intracranial tumors encompass a rare and heterogeneous group of entities. Owing to advances in molecular technology, an increasing variety of EWSR1 fusion partners have been described. The reported EWSR1 gene fusion partners in CNS mesenchymal and primary glial/neuronal tumors comprise CREB1, CREM, PATZ1, and PLAGL1 (2–6). EWSR1-PLAGL1 is a rare fusion partner with few cases reported in the literature (5–7). PLAGL1 is a member of the imprinted gene network (IGN), which is paternally expressed. Varrault’s work identified PLAGL1 as a transcription factor that coordinated the regulation of a subset of IGN genes and controlled extracellular matrix composition (8). The molecular mechanisms by which PLAGL1 participates in such diverse processes remain to be elucidated.

To the best of our knowledge, only three EWSR1-PLAGL1 fusion intracranial tumors were reported. One case was in the setting of a large-scale molecular study which also showed INI-1gene deficiency and lacked morphological and IHC description (7). In Sievers’s study, a high proportion (5/13) of cases were ependymomas, and all the cases showed positive expression of GFAP and negative to Oligo-2 and SOX10 (5). Lopez-Nunez’s study reported a case with a biphasic histopathology pattern which shared similarities with our reported case. We are also in agreement with their speculation that the biphasic feature might be related to the “dual effect” of PLAGL1 in promoting the proliferation of the primitive cells (6). However, there are still some notable differences. The former case was identified as a glioneuronal tumor with two tumor components: the large ganglion cell-like tumor cells with expression of NF and Syn; the small uniform tumor cells with expression of GFAP.

Our reported case showed no glial cell differentiation, such as GFAP and Olig2, while Vimentin was strong expressed for all the tumor cells. These results were not supported the diagnosis of ependymoma, glioneuronal tumor, or other gliomas. So it is initially presumed to be an atypical central neurocytoma because Syn was strongly expressed in all the tumor cells. Moreover, we found a unique tumor feature with a variable biphasic epithelioid differentiation which was not previously reported. The immunohistochemistry results further confirmed the unique feature of Cytokeratin expression in epithelioid differentiated tumor cells. Both primary and recurrent tumors were well-defined, with mild atypical tumor cells in a patchy growth pattern. Epithelioid differentiated tumor areas were enlarged in the recurrent tumor. Besides, the Ki-67 index was increased compared with the primary tumor. It implies epithelioid transformation might contribute to long-term tumor progression in this morphological special EWSR1-PLAGL1 tumor.

Here, we reported a recurrent intracranial EWSR1-PLAGL1 rearranged tumor with unique biphasic epithelioid differentiation and immunohistochemistry features which differed from the previously reported cases. NGS showed TERT promoter mutation. Our study provides further insight into intracranial tumors with EWSR1-PLAGL1 fusion, and future studies, particularly for the biphasic differentiation and the role of TERT promoter mutation might be needed to clarify this rare rearranged tumor.

## Data Availability Statement

The datasets presented in this article are not readily available because original sequencing result has been showed in **Figure 4A**, and follow-up studies are still going on. Requests to access the datasets should be directed to corresponding author Professor Kun Mun (mukun@sdu.edu.cn).

## Ethics Statement

The studies involving human participants were reviewed and approved by the Ethics Committee of Qilu Hospital of Shandong University. The patients/participants provided their written informed consent to participate in this study. Written informed consent was obtained from the individual(s) for the publication of any potentially identifiable images or data included in this article.

## Author Contributions

A-yX performed microscopy and data analysis. W-wY helped with the interpretation of data and manuscript writing. Y-lL, N-nS, and X-mH provided helpful advice for the interpretation of data. S-xW helped with the microscopy data. KM performed microscopy, data analysis and wrote the manuscript. All authors read and approved the final manuscript.

## Funding

This work was supported by the National Natural Science Foundation of China (No. 81572594 and 81902884).

## Conflict of Interest

The authors declare that the research was conducted in the absence of any commercial or financial relationships that could be construed as a potential conflict of interest.

## Publisher’s Note

All claims expressed in this article are solely those of the authors and do not necessarily represent those of their affiliated organizations, or those of the publisher, the editors and the reviewers. Any product that may be evaluated in this article, or claim that may be made by its manufacturer, is not guaranteed or endorsed by the publisher.
