# Supplementary material for: Rare Recurrent EWSR1-PLAGL1 Rearranged Intracranial Tumor With Biphasic Epithelioid Differentiation: One Case Report With Literature Review
Source: Front Oncol. 2022 Jul 14;12:938385. doi: 10.3389/fonc.2022.938385 (PMC9329790; doi:10.3389/fonc.2022.938385)
Supplement: Supplementary file 1 [file DataSheet_1.docx]

**Supplementary Figure**


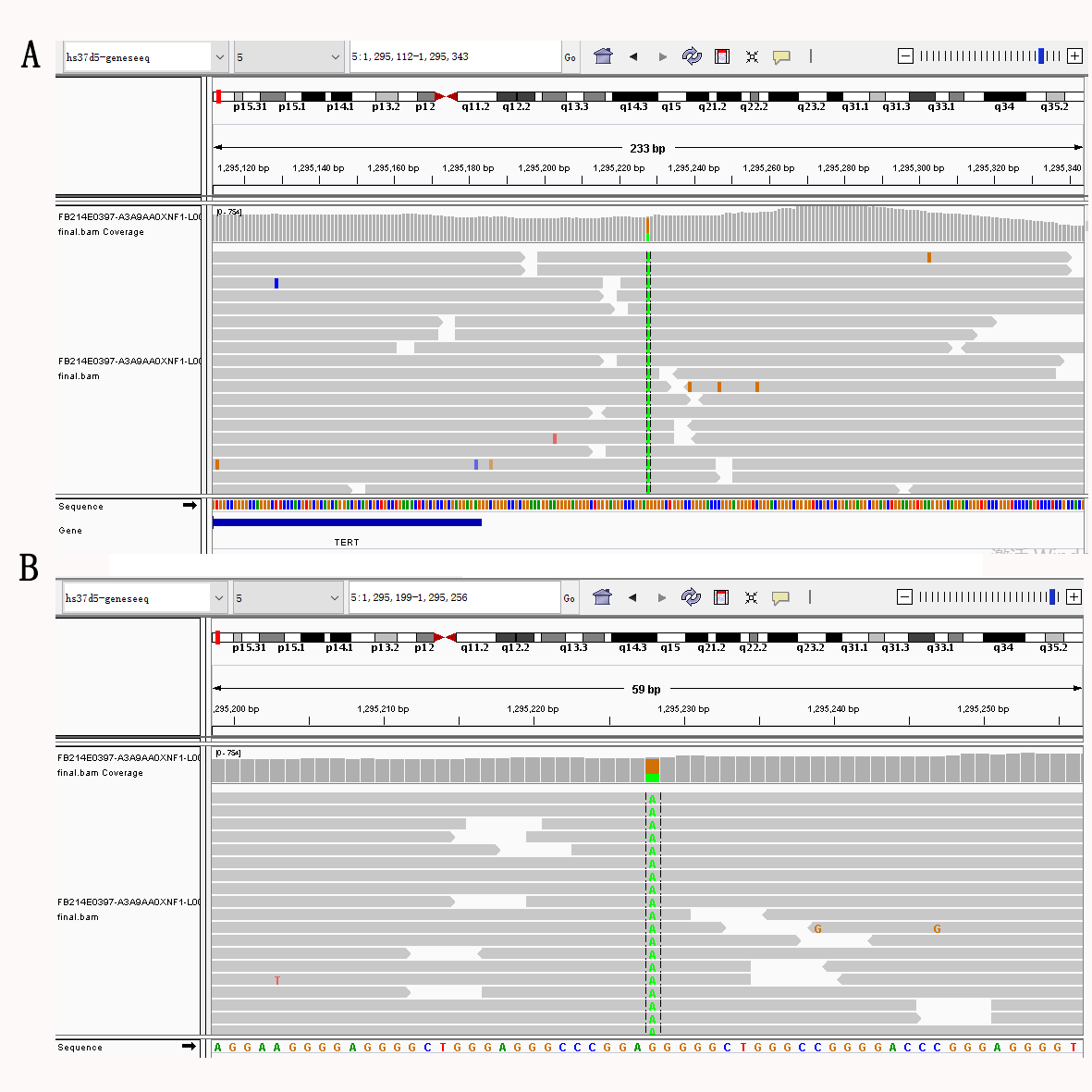


The mutation site of TERT promotor (c.-124C>T）was showed. The mutation is in the upstream of the start codon of TERT gene (A). The mutation gene site was G>A (B).

**Supplementary Table**

The panel of 82 genes

| AKT1 | CDK4 | | ERBB2 | GSTP1 | KRAS | MYCN | PKHD1 | SMO | | |  |
| --- | --- | --- | --- | --- | --- | --- | --- | --- | --- | --- | --- |
| APC | CDK6 | | ERCC1 | GSTT1 | LRP1B | NF1 | PMS2 | STAG2 | | |  |
| ARID1A | CDKN2A | | ERCC2 | H3F3A | MDM2 | NF2 | PTCH1 | TERT | | |  |
| ARID1B | CDKN2B | | FAT1 | HRAS | MDM4 | NOTCH1 | PTEN | TP53 | | |  |
| ARID2 | CDKN2C | | FGFR1 | IDH1 | MEN1 | NOTCH2 | PTPN11 | TOP1 | | |  |
| ATM | CHEK2 | | FGFR2 | IDH2 | MET | NOTCH3 | RB1 | TSC1 | | |  |
| ATRX | CREBBP | | FGFR3 | KDM6A | MLH1 | NRAS | ROS1 | TSC2 | | |  |
| BRAF | CTNNB1 | | GNAQ | KDR | MSH2 | PDGFRA | SETD2 | XRCC1 | | |  |
| BRCA1 | DAXX | | GNAS | KIT | MSH6 | PIK3CA | SMARCA4 | YAP1 | | |  |
| BRCA2 | EGFR | | GSTM1 | KMT2C | MYC | PIK3R1 | SMARCB1 | UGT1A1 | | |  |
| MGMT | TERT | | 1p19q |  |  |  |  |  | | |  |
|  | |  | | | | | | |  |  | |
